# A Contagious Other? Exploring the Public’s Appraisals of Contact with ‘Mental Illness’

**DOI:** 10.3390/ijerph17062005

**Published:** 2020-03-18

**Authors:** Daniel Walsh, Juliet Foster

**Affiliations:** Institute of Psychiatry, Psychology & Neuroscience, King’s College London, London SE5 8AB, UK; juliet.foster@kcl.ac.uk

**Keywords:** public understanding of science, implicit stigma, risk perception, embodied cognition, affect, contact theory, mixed-methods, social representations, mental illness

## Abstract

Mental illness has recurrently been found to be Othered by the lay public, although few researchers have examined the affective and implicit processes involved. To explore this, we triangulated facial electromyography (EMG), self-reports, and individual interview data, finding participants to Other mental illness, a process that involved disgust, fear and pity. Furthermore, mental illness was considered to have the potential to permeate, posing a contagious threat. This research highlights the need to fully explore the forms of understanding, which maintain mental-health related stigma, including beliefs about contamination, and the implications this may have for the design of anti-stigma campaigns.

## 1. Introduction

Contrary to the expectations of some researchers (e.g., [1,2]), forms of common-sense thinking about mental health problems, including superstitious belief systems, are not necessarily displaced by increases in mental-health related literacy [3,4,5]. Rather, traditional forms of understanding, such as ‘madness’ being ‘contagious’, can often co-exist with more “modern” forms of knowledge [6,7,8,9], although this may be outside of conscious awareness [10,11]. Despite theories of science literacy being widely challenged, anti-stigma campaigns typically continue to assume a positive linear relationship between mental-health related literacy and stigma-reduction. This has been found to have some unintended consequences: for example, subscribing to biomedical models of causation has been associated with more negative appraisals of contact with someone with a mental illness [5,12,13]. In response, this research examines the forms of knowledge held by the public, paying particular attention to the implicit and affective processes that may sustain the stigmatization of mental illness [4,8,10].

### 1.1. Stigma

Mental health-related stigma is a ‘wicked’ problem in the UK, operating at structural, interpersonal, and intrapersonal levels [13]. Despite high profile campaigns to change attitudes towards mental health problems (e.g., Time to Change in England; See Me Scotland), some theorists suggest mental illness remains Othered [4].

Broadly, Kalampalikis and Haas [14] define the Other as a belief that guarantees, orchestrates or institutes difference, something that may often involve descriptions of being uncommon, non-familiar, strange and fundamentally “not-me”. To construct a group as Other is a practice of ‘stigmatic thinking’ [15]. Since Goffman’s classic formulation of stigma [16], the public has been found to systemically Other those perceived to have a mental illness, often by constructing them as different, unpredictable and violent [3,8,17,18,19].

An under-developed literature suggests this Othering may involve implicit beliefs of contamination [7,20,21]. As far as we are aware, no studies have directly examined the possible affective processes involved in a belief of mental illness as contagious.

Before describing the design and results of the study conducted to begin to fill this gap, we will first review the history of public understandings of mental illness and ‘madness’. We will highlight an under-theorised theme concerning practices of separation, often involving beliefs of contamination. Next, we will consider the methodological challenges of this area, proposing that using theories of embodied cognition—specifically appraisals of disgust—may reveal new insight into the possibility that the public implicitly holds beliefs that mental illness is contagious.

### 1.2. Public Understandings of ‘Madness’ and Mental Illness

A key theme framing public debates about mental illness has been arguments in favour and against inter-group contact [18,19]. These debates often express implicit beliefs, including beliefs of contamination [7,10,19], and are possibly rooted in a historical fear of ‘madness’ [8].

Since the late medieval period, there has been public discussion about the need to limit contact, especially intimate contact, with the ‘mad’—supposedly both for the perceived health of the broader society and the perceived wellbeing of the ‘mad’ themselves [21,22]. This would later be institutionalised in public perception through the location and design of asylums and psychiatric hospitals [23]. There was a near obsession with ventilation in the design of these buildings, reflecting the widely held belief that high-quality air could cleanse ‘mental impurities’, cementing a belief in the need to prohibit certain forms of contact [21,23].

We continued to see some aspects of this into the 20th century. For example, Jodelet documented a community in rural France in which patients from a local hospital lived as ‘lodgers’ in the homes of local families [8]. She found that notions of contamination structured lodger-host interactions, although host families explicitly denied a belief that mental illness was contagious [8]. To minimise a perceived risk of ‘pollution’, host families avoided sharing crockery and cutlery with their ‘mentally ill’ lodgers, washing laundry together or handling liquid forms of psychiatric medication [8].

By the mid-20th century, de-institutionalisation across western Europe was well underway [4]. This increased scope for contact was matched by a developing moral panic, maintaining the representation of mental illness as Other [20]. Instead of challenging the perceived risk posed by contact with mental illness, this risk was reimagined as a continual threat experienced in daily life [19].

Although public understandings of ‘mental illness’ or ‘mental health problems’ as a unitary and unified term has recurrently been found, differentiation in understandings is also evident between perceived groups of mental illnesses [3,17,24]. Such studies find that schizophrenia is perceived as the prototypical violent threat, held in comparison with depression, which is typically seen as relatively more familiar and comprehensible [5,17,24,25]. Moreover, biological factors more than social factors are suggested as potential causes and this correlates with a greater desire for social distance [5,24,25]. Furthermore, recent evidence suggests that depression is believed to be more communicable than schizophrenia, although there were limited differences in willingness for interaction [26]. It is now commonplace in examinations of the public’s attitudes and beliefs about mental illness to compare depression and schizophrenia [12]

### 1.3. Embodied Cognition

Although there is evidence to suggest beliefs of contamination may be involved in public understandings of mental illness, these may be implicit [7,8,20], presenting a methodological challenge [10,11]. In response, in an attempt to access these implicit meanings, we will draw on theories of embodied cognition.

Relevant to our current discussion is the so-called ‘magical law of contagion’ [27,28,29]. This refers to the belief that groups can leave their ‘essences’ on objects, spreading infection. For example, clothes worn by HIV-positive individuals were found to be seen as taking on the contagious profiles of their wearers [27]. Additionally, this ‘magical law of contagion’ is not restricted to objects that have physically touched a perceived contaminate or out-group, but also applies to objects near a perceived contaminate or semantically associated with it [28].

Aversion to contact with stigmatised out-groups is found to involve appraisal of negative affect and embodied experiences of disgust [29,30]. Disgust has been understood as a ‘guardian of the mouth’ [31], designed to reduce exposure to ‘pathogens’ [20]. Indeed, the levator labii superioris, a facial muscle close to the mouth opening, is increasingly found to be involved in appraisals of disgust with only a limited number of studies failing to find an effect [32]. However, this ‘pathogen’ is not restricted to a biological form of infection. Rather, disgust is suggested to be a socially elaborated emotion, elicited by objects or beliefs considered socially undesirable, and is often directed towards groups constructed as Other [27,28,29,32,33].

Research into disgust and Otherness has also paid attention to disgust-sensitivity, which is considered to be an inter-individual measure of the predisposition to experience disgust. Disgust sensitivity is consistently correlated with forms of stigma, including homophobia, islamophobia and depression-related stigma [30].

### 1.4. Summary

In the remainder of this paper, we examine the cognitive and affective psychological mechanisms involved when simulating contact with mental illness. We examined these using a student sample (N = 36), employing convergent measures in a mixed-methods triangulation design. The participants’ current levels and forms of contact with mental illness, the beliefs they held about mental illness, how they affectively responded to mental illness, and whether their responses were differentiated by disorder label and disgust-sensitivity were measured.

This research extends previous findings by directing attention to (1) the affective experience of contact; (2) the beliefs engaged during contact, including fears of contamination; (3) how the experience of contact is differentiated by disorder label, and finally by (4) suggesting a symbolic protective function of holding stigmatising beliefs towards mental ill-health.

#### Research Highlights

- Mental illness is Othered and engaged fears of contamination.
- Constructions of mental illness elicited appraisals of disgust, fear, pity, and compassion.
- Appraisals of mental illness are differentiated by disorder label between schizophrenia and depression.
- Public health anti-stigma campaigns need to tackle implicit prohibitions around intergroup contact.

## 2. Materials and Methods

### 2.1. Participants

Sample size was calculated a priori using G*Power-3. A sample of 36 is sufficient to have a 95% probability of finding an effect size f = 0.25 for modelling in repeated-measures within-factors designs, containing 2 between-subjects levels, 4 measurements, a correlation of 0.5 among measures and a non-sphericity correction of 1. Samples of this size have been found to be sufficient to examine within-individual differences in facial electromyographic activation using vignette-based simulationist designs [34] and the public’s appraisal of contact with schizophrenia [35]. It was also felt that theoretical saturation of qualitative elements was likely in a sample of this size [36].

In 2017, the study was advertised to all members of two universities within the same town as exploring understandings of health. Participation was confined to a student sample for two reasons: first, this common background allowed for the construction of a procedure relevant to the participants shared context (see below); second, it has been suggested that student populations are of particular interest when it comes to stigma reduction programmes [12]. All participants were required to be aged over 18 years, with normal or corrected-to-normal vision and hearing, and no history of mental illness. An initial sample of 40 participants was recruited (Table 1). However, four participants were excluded from the final analysis: one for not being a registered student, one for not being able to understand the experimental procedures and two because of malfunctions in the recording equipment. The final sample comprised 36 participants (female: 23 (63.9%); male: 13 (36.1%)), of which 5 (14%) studied psychological or health sciences. The majority of the sample recruited was in early adulthood (M = 24; SD = 5); and reflected a broad range of subjects and nationalities. The implications of this will be considered later in the paper.

### 2.2. Materials and Procedure

Approval was obtained from the University Psychology Research Ethics Committee (PRE.2016.083). All participants were tested alone and sessions lasted up to 1 h. On arrival at a university laboratory, the first author orally explained the study to participants, who had already received an information sheet. The full research questions of the study were not divulged at this stage. Participants then signed a consent form and were then prepared for facial electromyography (fEMG). Following the standards set by Fridlund and Cacioppo [37], the researcher placed three silver/silver chloride (Ag/AgCl) electrodes contralateral to participant handedness: one on the forehead, one 1 cm lateral to the baseline of the ala nasi and one 0.5 cm below the alar curvature point. The skin was prepared with an abrasive scrub, and a conductive electromyography (EMG) gel applied. The electrodes were connected to a BIOPAC 150 acquisition unit, using a wireless EMG amplifier. The procedure then began, consisting of three sequential stages: questionnaire, vignette presentation and interview. After all conditions were completed, the participant was fully debriefed, and the full details of the study discussed. Given that the full research questions were not revealed to participants prior to the study, all participants were given the opportunity to withdraw their data from the study at this point; no participant chose to do so. 

#### 2.2.1. Abbreviations

DSDisgust SensitivityPCMIPrior Contact with Mental IllnessRIBSReported and Intended Behaviour ScaleGEWGeneva Emotion WheelHCNVHigh-Control Negative-ValenceLCPVLow-Control Positive-Valence

#### 2.2.2. Questionnaires

Questionnaires recording general demographic information, disgust-sensitivity (DS) and prior contact with mental illness (PCMI) were completed by participants. General DS was operationalised using the Disgust-Scale Revised [31,33], which has been found to be predictive of avoiding disgusting objects/situations [31,33]. This disgust-scale (DSR) is a self-report personality scale used to measure individual differences in disgust. We included this scale to examine how participants’ appraisal of the vignettes varied by trait-level disgust sensitivity. After excluding any participants who failed inattention ‘catch’ questions, individual participant responses to the DSR questions were summed, and reverse coded where appropriate. A good internal consistency was found (α = 0.85).

Form and frequency of prior contact with mental illness were measured using a subsection of the Reported and Intended Behaviour Scale (RIBS) [38]. This is the standard instrument for public mental health anti-stigma evaluation, often used in conjunction with other attitudinal- and behaviour-related measures [39]. Participants completed this sub-section of the RIBS questionnaire to allow researchers to generate descriptive statistics detailing participants’ self-reported frequency and form of prior contact with someone with a mental health problem (Table 2). Additionally, this questionnaire was included to explore possible quantitative relationships between frequency of prior contact and the affective experience of imagining contact with someone with a mental health problem. Descriptive statistics generated on this subsection of RIBS questionnaire revealed roughly half the sample had one or no forms of exposure to mental health problems (47.2%), and accordingly were grouped as low PCMI. Conversely, participants reporting two or more forms of exposure to mental health problems were grouped as high PCMI.

#### 2.2.3. Vignette Presentation

During this stage, each participant was sequentially presented with four vignettes, the order of which was randomised and counterbalanced across the sample. All participants responded to all four vignettes. Vignettes were developed with reference to the literature and research questions and piloted on 5 people (Appendix A & Appendix B). We exposed all participants to all the treatment conditions for three main reasons: (1) fEMG has continually been noted to be limited by significant within inter-subject variability in facial morphology and physiology (for a review see [40]), suggesting a within-person analysis may be more effective in reducing these sources of bias; (2) recent studies using this form of design highlight how subtle linguistic differences are useful for exploring within-individual differences in facial activation whilst reading moralised stories [34]; (3) qualitative comparisons between perceived groups of mental illness can be useful for examining the implicit aspects of its representation (6).

Before reading each vignette, the participant took a sip of water from a fresh glass placed in front of them and was asked to hold it in their mouth. Each vignette asked the participant to imagine that they were working on a project with a classmate with whom they were unfamiliar. In three of the conditions, the participant was given an extra piece of information about their classmate. They were told that they had been informed that their classmate had either: 1. depression, 2. schizophrenia, 3. a common cold or 4. no-added medical description. The third and fourth categories were included for comparison purposes. The vignette continued by asking the participant to imagine that both they, and their classmate, were drinking a cup of tea, when they realized they had accidentally been drinking from their classmate’s mug. Participants were asked to imagine that the water they were holding in their mouth was the tea from their classmate’s cup.

Throughout this process, differences in electrical potential at the levator labii superioris were measured using fEMG. Gain was set at a sample rate of 1000/s, and the initial signal was amplified and filtered online at a band pass of 5–1000 Hz. The signals were digitised online, calculating a mean EMG root square value using AcqKnowledge v4.1 software. Concurrent screening for frequency noise was applied using a notch-filter of 50 Hz.

After reading each vignette, still holding the water in their mouth, each participant completed the Geneva Emotion Wheel (GEW) [41]. The GEW is structured in a circular format asking the participant to rate whether, and with what intensity, they felt 20 pre-selected emotions whilst reading the vignettes. GEW measures self-reported appraisals along 3 dimensions: valence, control and arousal. It is organized to fit a 2 × 2 grid of valence and control, which is further differentiated by arousal level [41]. Control represents the degree to which the individual considers themselves able to respond adaptively to the threat [41], whereas valence is the degree of instinctive positivity/negativity according to goal conduciveness [41]. Arousal represents the strength with which an emotion is experienced [41]. The GEW allows both a comparison between scenarios on groups of emotions, whilst also facilitating measurement of specific individual emotions.

Once the GEW was completed, the used materials were taken away, and a fresh cup of water, straw, and GEW were placed in front of the participant before the next vignette was presented. Between conditions, the participant was presented with a series of neutral images (N = 60) to return them to a resting arousal rate. Each image was presented for 5 s. Images were pre-validated as neutral in valency and arousal [42].

Offline, to prepare fEMG data for testing, following the recommendations set by van Boxtel [43], a ratio score was calculated with a variable participant resting rate, comparing mean activation pre- and post-stimulus onset. As pilot testing revealed participants on average took 20 s to read each vignette, this was used as the upper limit for the four mean EMG amplitudes recorded post-stimulus onset. A 5 s interval was used to avoid artefact effects associated with movement. This meant that each participant had four mean ratio scores, one for each treatment condition. Mean ratio scores were transformed by 1/log10 to meet assumptions of normality for parametric testing.

Individual self-reported emotions were summed and grouped, reflecting underlying GEW dimensions [41]. Emotions were grouped according to control and valence: High-Control/Positive-Valence (interest, amusement, pride, joy, pleasure); Low-Control/Positive-Valence (LCPV: contentment, love, admiration, relief, compassion); Low-Control/Negative-Valence (sadness; guilt; regret; shame; disappointment); High-Control/Negative-Valence (HCNV: fear; disgust; contempt; hate; anger).

#### 2.2.4. Interviews

After all four vignettes had been presented (Appendix A); semi-structured interviews were used to develop a depth-account of participants’ appraisal of the vignettes, focusing on their understanding of mental health, and norms around contact with mental ill-health. An interview guide (Appendix B) was used to ensure comparability across the sample. It was applied flexibly, probing participant understandings as appropriate. The researcher strove for a collaborative style of conversation, using reflection and summarization to provide a ‘depth’ account of participant representation, without directing responses [44]. The first author conducted and audio-recorded the interviews. Additionally, they transcribed verbatim the audio-recordings’ verbal features. 

#### 2.2.5. Triangulation

The study employed triangulation combining qualitative and quantitative measures [44]. Each measure is considered to provide partial insight into the question of interest and each measure is considered both individually and in relation to others. The analysis consisted of four stages. We will first discuss further details on the methods used for qualitative and quantitative forms of analysis and then explain the overall structure of the analytic strategy taken.

#### 2.2.6. Quantitative Analysis

To quantitatively evaluate appraisal, repeated-measures analysis of variance (ANOVAs) and Bonferroni’s procedure were used, comparing within-individual differentiation in self-reported emotions, and differentiation in levator labii superioris activation. To assess equality in variance between all combinations of the conditions, Mauchley’s test of sphericity was applied. If violated, degrees of freedom were corrected using Greenhouse–Geisser (ε ≤ 0.75), and Huynh–Felt adjustments (ε ≥ 0.75).

#### 2.2.7. Qualitative Analysis

Thematic analysis was employed for qualitative analysis [44]. Latent and semantic themes contained within participant narratives were coded both inductively and deductively. Codes were generated iteratively, moving backwards and forwards through the process of open, axial, and selective coding, progressively generating thematic domains and categories [44]. Particular focus was given to participant appraisal of vignettes, considering the implicit affects and cognitions used to make sense of mental health and ill-health. Coding ended when no new codes or themes were generated [36]. An initial coding book was produced by the first author. To establish the trustworthiness of the study, a data audit was completed [44] with the second author. This included reviewing the study’s research aims and design, re-examining the coding book using raw participant transcripts, and critically reflecting on possible biases present in the study. Furthermore, two key quality indicators were used in this project: confidence and relevance [44]. Confidence is provided by using triangulation, as it ensures reflexivity throughout the whole project [45]. Furthermore, triangulation requires the researcher to both acknowledge the limitations of each methodology utilised, but also negotiate the tensions between different results [45]. Thus, triangulation was used both in developing the analysis plan, and in its interpretation. Relevance was ensured by ‘surprise-value’. Specifically, this was ensured by coding both inductively and deductively, remaining rooted in the research question, but being open to new insights [44]. 

#### 2.2.8. Analytic Strategy

(1) Validity of Measures

First, we evaluated the validity of the experimental procedures. To do so, we examined how participants interpreted the vignettes, as described during their follow-up interview. Open coding of the transcripts found that participants considered the vignettes to describe a believable context and that they elicited a mixture of affects and cognitions. In particular, participants’ experiences of simulating contact with mental illness fitted with a ‘magical law of contagion’ [27], with participants expressing essentialist beliefs about essences whilst imagining sharing liquids [26]. In opposition, for the common cold, participants’ beliefs about infection risk were localised to direct points of shared contact, and participants held developed beliefs about mechanisms for infection, matching previous literature [26].

However, open coding also revealed the no-added medical description vignette to be an unreliable control, as there was considerable variation in its interpretation. For some participants, this condition was understood as intended, eliciting emotions of neutral valence and low-arousal. However, for others, lack of extra information to describe their imagined classmate was anxiety-provoking. Indeed, some believed their partner to have a hidden illness, a belief they found more concerning than ‘knowing’ about a condition, with some participants describing their partner as a stranger with undescribed extra illnesses and even a sexually transmitted infection.

*“initially, when I read the one where you just used the other persons mug, I thought okay, not too big a deal, a bit gross, but you know, okay. But then I started thinking about it, wondering what I hadn’t been told about them, that’s when I started to worry” (p. 17).*

As P.17 describes, interpreting this vignette was a non-linear process, where P.17 experienced multiple beliefs and affects at different times as their interpretation of the vignette evolved. Considering the complexity and variability in interpreting this condition, this vignette was found to be an inappropriate control condition for modelling differences using general linear models. As the study lacked a suitable control, quantitative analysis will only be used to explore differentiation between the vignettes relating to depression and schizophrenia. However, the implications of this will be fully considered. 

(2) Prior and Projected Contact with Mental Illness

Form and quantity of prior and projected contact with mental illness were calculated using the Reported and Intended Behaviour Questionnaire. Additionally, interview data was used to enrich this, highlighting the multiple forms of prior contact.

(3) Experiential Aspects of Contact with Mental Illness

To understand the experience of contact, first we considered the broad themes participants verbally expressed when making sense of mental illness. Next, to draw out the affective aspects of contact, we quantitatively analysed possible differences in activation at the levator labii superioris and self-reported emotions. Additionally, during the axial coding, we considered the thematic binaries participants drew upon to compare contact with depression and contact with schizophrenia. Last, to enrich quantitative results, we selectively coded for the thoughts and feelings participants reported experiencing when sharing the glass of water.

(4) Overall Triangulation

As previously described, experiences of contact with stigmatised groups—as inclusive of individually experienced affects, values, and motivations—are organised by the wider social norms and cultural influences within which the individual is situated [25]. Accordingly, in this stage we triangulate towards an explanatory account of participants experiences of contact.

## 3. Results

### 3.1. Prior and Projected Contact with Mental Illness

Overall, most participants reported some form of prior contact with someone with a mental health problem; with only one in five reporting no prior contact. However, contact came in multiple forms: roughly half the sample reported a close friend with a mental health problem (Table 3), which is broadly comparable with national average in 2017 [39]. Expanding our understanding of forms of contact, interviews revealed participants as engaging with mental health and ill-health in multiple ways. These ranged from mediated contact through media sources (TV, movies, newspapers, social media and plays), to in-person experiences, predominantly with close family and friends.

Focusing on the more symbolic forms of contact, participants showed an aversion to sharing personal objects with someone perceived as having a mental health problem, often centring on the sensitive points of personhood. For example, P.31 describes avoiding contact through objects that have links with the head:

*“I would say anything that has connections, or some sort of links with the head, … pillow, crash helmet … I think it is a feeling of how dangerous can it be to pass on to me… how dangerous can it be if I talk to this person too much for a long time, how can I be somehow contaminated by his ideas or somehow influenced by something that I don’t know”. (P. 31)*

*Similarly, P.12 expresses concerns over: “their computer, I mean that is kinda a reflection of the kind of person they are, and like probably their condition influences what they do.” By avoiding sharing a computer, she maintains her distance from the perceived personal characteristics and behaviours of someone with a mental health problem.*

A self-protective element may be involved in distancing behaviours and prohibitions of contact. For instance, P.9 also expresses concerns about sharing a computer: “I don’t want to share clothes, or a computer maybe. I think my computer has so much on me in it ... you don’t know what they’ll do”. Here, by avoiding a shared computer, she symbolically minimizes a perceived risk; a risk of exposing herself. A self-protective element may also take on group dimensions, with participants moving flexibly between first and third person pronouns: “they have fear that it’s going to pass them. They dont want to be like them. I think I just want to be in the normal group, and not the not-normal group.” (P.34). By minimising close forms of contact, P34, aligned herself with an in-group; the ‘normal’ group, and distanced herself from Othered out-group.

### 3.2. Experiential Aspects of Contact with Mental Illness

Overall, across all conditions, sharing a mug with classmate was generally considered to present some degree of risk. In the common cold condition, this risk was primarily considered to present a germ-based form of infection risk.

*“If you’ve seen somebody with a cold sneeze onto something, … then obviously there would be germs on there” (p. 3)*

*“For the cold, I think I would have answered very differently if I had to use a spoon that someone else had used. Because with the mug, you would have had to place your lips exactly on the spot where the other people put it.” (p. 23)*

Diverging from how the participants experienced contact with someone with a mental illness, as described in Section 3.1, students with a common cold were not represented using a group-based language, the possessions of someone with a common cold were not anthropomorphized to reflect undesirable personality characteristics; perceived risks of contact were localised to specific points of shared contact. In contrast, participants typically constructed mental illness as different and abnormal, or in other words, as ‘Other’. Indeed, whilst across all conditions participants expressed a sense of having violated a social norm by drinking from a classmate’s mug: when the classmate was perceived as having a mental illness, this social norm took on new meanings:

*“You don’t know what to do now, in that situation, it’s basically like ‘ahhh’. I feel like I could feel the difference in kinda that feeling. It was different categories. It was really something that is like like ‘oh’, they are more different, like psychologically” (P. 19)*

P.19 felt he had crossed a boundary and had entered a different ‘psychological’ category, an experience that resisted labelling, drawing instead on ‘non-words’ to convey his anxiety around contact. In more extreme accounts, a concern about sharing with someone perceived as psychologically different drew on a language of abnormality. For instance, P.34 states: “they don’t think as normal people … I want to stay away from this person”.

Imagining contact with mental illness elicited a range of appraisals, the affective components of which sometimes involved experiences of disgust and fear. In the most extreme accounts, the perceived threat posed by contact with mental illness engaged a visceral response:

*“Is there emotions ‘I just want to escape’? I just want to get away, I just want to stop thinking this. I feel sickness in my stomach. This is strong, I feel I want to eugh [pretends to vomit]. This is a strong emotion … disgust, yeah. Extremely disgust!” (P. 9)*

However, not all participants experienced this form of appraisal. Indeed, there were also expressions of compassion: “My emotions were different just because I was imagining sitting with someone that had gone through something … and hoping they were okay.” (P.13). However, there was a fine line between compassion and pity: “I was a bit freaked out because they might [pause]. I don’t want to say they are crazy, but like [pause]. Well I felt bad for them in both cases” (P.10). P.10 makes sense of mental health problems through an image of the Other as ‘crazy’, something discrediting, which elicits sadness and fear. Drawing participants’ descriptions together suggests that an idea of difference associated with mental illness engaged an embodied form of appraisal, ranging from being uncomfortable to a concerted desire to flee, and from compassion to pity.

Within a unified appraisal of mental illness, differentiation in the degree of affective arousal elicited by contact with mental illness was found between disorder labels. Specifically, schizophrenia was found to elicit more HCNV emotions [F(1, 35) = 5.264, *p* = 0.028, ηp^2^ = 0.141], and fewer LCPV emotions [F(1,35) = 17.513, *p* = <0.001, ηp^2^ = 0.361] than depression (Table 4). Additionally, schizophrenia engaged greater activation at the levator labii superioris relative to depression [F(1,35) = 4.53, *p* = 0.04, ηp^2^ = 0.118] (Table 4). No individual differences were found according to DS or PCMI. As differentiation was only found in the emotion groups elicited by a unified image of mental illness (fear, disgust, compassion), appraisal of contact seems to be negotiated by disorder label, whilst remaining constrained within overarching beliefs about mental illness. Furthermore, as differentiation was found in facial activation at the levator labii superioris—an index of disgust-related affect—this suggests that the body is involved in appraising contact with mental illness, and appraisal may involve an affective process, which could be outside conscious awareness.

An examination of participant narratives provides some insight into the different levels of arousal found between depression and schizophrenia. Overall, participants divided forms of mental illness according their perceived unpredictability, familiarity, comprehensibility, symptomology and cause.

In general, schizophrenia was perceived as the prototypical violent Other. For example, as P.21 describes: “in schizophrenia there is an—[pause]—like a bit unknown about, not unknown, unpredictability—[pause]—and that is frightening”. Indeed, participants felt their only contact with schizophrenia was through the media (TV, movies and newspapers) and was felt to be ‘invisible’ (P.10) in student communications. As P.41 explains: “I don’t really know people with [schizophrenia] … they’re a bit everywhere in the movies, books, as crazy, as wanting to kill” (P.41). In this low state of perceived in-person contact, schizophrenia retained an image as different and violent. However, whilst considered invisible in their immediate environment, its media representation rendered it immediately available in their imagination.

In contrast, depression was felt to be more familiar and understandable. As P.20 explains: “maybe because I know people with this problem… I’ve never been really depressed but sometimes I’m a little bit low, so I can understand them better, and I think I feel close to them [pause], closer to them.” Depression was predominantly considered to be a disorder of feeling ‘low’ and ‘emotional’, a symptomology the participants felt they could understand and with which they could empathize, contrasting with the image of schizophrenia as incomprehensible. As P.37 explains, firstly in relation to schizophrenia: “they have things going on that I can’t comprehend or see, so I’m not gonna press or cause any extra difficulty … I feel I just understood the depression more”. Here, an image of schizophrenia as incomprehensible is linked to behavioural restriction, limiting the scope for intimate interpersonal contact.

Descriptions of compassion towards depression were often matched by descriptions of intimate prior contact. For example, P.21 argues her responses to the vignettes to be contingent upon having close friend with depression: “I don’t have any close friends with schizophrenia, but I do have a close friend with depression… I had more like compassion, because I was thinking of my friend” (P.21). Conversely, only two participants reported having in-person contact with schizophrenia. Triangulating this with PCMI suggests a high overall self-reported prior contact to be skewed towards perceived contact with people experiencing depression not schizophrenia. However, one must be cautious about interpreting the positive effects of prior contact, as participants’ descriptions of compassion also often expressed pity. For example, as P.26 describes: “depression, you imagine them as a victim … and you want to feel compassionate”. She highlights how expressions of compassion are considered socially desirable, and links this to image of victimhood; arguably an elicitor of pity. Furthermore, it is important not to overstate the perceived differences between depression and schizophrenia. Indeed, the differences were relative not absolute:

*“If they are in depression, I’m afraid they will do something aggressive, especially in private room. Just speak to someone in depression, she just start crying or screaming or lots of negative complaints” (P. 9)*

*“People with depression, or being prone to be sad or over-upset by small things, their emotions are quite volatile …. Something like this could easily upset them.” (P. 27)*

Expressing an image of depression as a proclivity for sadness and volatility, some participants engaged in a unified appraisal of mental illness as Other, contact with whom is risky. Moreover, for some, this perceived Otherness involved fears of contamination:

*“People with depression, or being in prone to be sad or have sad attitudes, or negative attitudes, I feel they transfer part of that to myself, and I don’t feel comfortable. I don’t despise them, but it makes me feel a bit upset or angry, because I feel how all that negative feeling is taking, transferring on to me” (P. 15)*

Here, P.15 expresses a belief that negative attitudes—a symptom she considers constitutive of depression—have the capacity to permeate. In response, she experiences anger towards this person. However, for others the anger was self-focused: “with depression, I felt more sorry, more angry at myself for doing it. Thinking this could have upset them”. Expressing an implicit belief that depression renders a person vulnerable, she describes anger towards herself for potentially upsetting them.

### 3.3. Triangulating the Data

Hitherto, as shown, contact with mental illness was experienced as ‘risky’, a risk differentially experienced by disorder label. The perceived risk elicited appraisals of disgust and fear; and images of mental illness as unpredictable, violent, unfamiliar. To understand why this ‘risk’ may have been maintained despite the length and intimacy of contact prior to the study, we will now layer the image of mental illness against participant normativity. Specifically, we propose appraisals that maintain distance between the Self and mental illness, reflect a desire for self-regulation and self-control, a desire potentially rendering the Self impervious to ‘risk’.

Participants engaged in a belief in health as an emergent and regulated process, rather than as a static or given state. Purposely, participants considered infection to be something that should be actively managed. For example, “I was worried for my own health … I’m like that, I back up my immune system” (P.14). P.14 does not see his immune system as an independent functioning process. Rather, he emphasizes its self-controllable aspects, considering it an object in need of nurture. This self-perception was opposed to participants’ perception of mental illness, which was constructed as a failure in self-control. As P.31 explains:

*“They would attach a degree of contagiousness to it. It’s the fear of losing control… or not knowing what you are doing. It’s dangerous, so better to seal it off” (P. 31)*

This participant describes a belief that an idea of contagion is embedded in public beliefs around contact with mental ill-health, linking it to a protective mechanism. That is, he suggests the public ‘seal off’ the perceived threat of ‘losing control’ by limiting contact with mental ill-health.

Not all participants were explicit about the connection between contagion and a failure in self-control. For others, the construction of mental illness as contagious was more subtle, through prohibitions around shared personhood (as previously described), or through its association with sexually transmitted diseases. For example, P.17 states: “after depression and schizophrenia I was expecting more like HIV, or something like this.” By engaging with mental illness through the same framework as HIV, she expresses the shared social meanings they hold in the public imagination. An association charged with emotion for P. 36.

*“Mental like, the people not like us, abnormals… normal is like, we can talk really … we think like human things, don’t imagine things, … they crazy, they abnormal, they take drugs, maybe like have HIV, or hepatitis A B C D. I do care, because I can be addicted to it” (P. 36)*

P.36 fluidly moves between a concept of abnormality and craziness, to sexually transmitted infections and addiction. She positions herself as “normal”, distinguishing herself from that which she sees as crazy. However, simultaneously, she suggests an identification with this abnormality, through her perceived ability to be ‘addicted’, though to what she leaves unclear. What we see is a construction of contact with mental illness as a threat to thinking ‘human things’; arguably a presenting a perceived risk of ‘losing the Self’.

## 4. Discussion

Participants’ understandings of mental illness highlighted a continued construction as Other, contact with whom is risky. The Othering of mental illness involved expressions about its perceived abnormality, unfamiliarity, and violence, fitting a traditional conceptualisation of the Other [14,46], as well as appraisals of disgust, fear, and pity.

Verbal constructions of mental illness as Other were concomitant with subtle social norms around contact, and affects of disgust and fear, both of which simultaneously functioned to maintain distance between the Self and the perceived Other. Specifically, participants avoided sharing objects associated with the purported individual with a diagnosis’ personhood, both to limit taking on their characteristics and to protect their vulnerability to those perceived characteristics.

This practice of appraisal held symbolic value for participants. By engaging in understanding mental illness as a failure in self-control, they delimited the boundary between themselves and the perceived threat. This fits into a wider literature of the Other, which finds infectious diseases to be attributed to marginalised groups, blaming their spread to the groups perceived ‘lack-of-control’ [11,47]. Indeed, across multiple domains of health and other issues evoking stigma, researchers find a close relationship between how participants from the general public construct their own identity (and that of those like them) and how they construct that of out-groups [10,11,46,47]. This is argued to be a motivated practice: first it renders the Self (and in-group) invulnerable to the perceived risk (mental illness); second it holds the out-group responsible for their own affliction [25,47].

Within a unified form of appraisal, contact with mental illness was found to be differentially experienced by disorder label. This practice in sense-making layered binaries of comprehensible/incomprehensible; familiar/unfamiliar; and predictable/unpredictable against a core image of mental illness as Other, in-keeping with previous literature [3,8,17,18,19]. Relatively, schizophrenia remained the quintessential Other, where a perceived lack of control rendered it a permanent threat, contact with which was perceived to be almost exclusively through the media, a source that prioritises implicit and explicit images of violence [18]. In comparison, depression was felt to be relatively understandable and familiar: intimate experiences with close family and friends allowed some participants to believe it was something they could empathise with. This fits with a wider literature in public understandings of science, which often highlights the dangers of asymmetric relationships between the media, the Self, and social others, and how this can sustain stigmatising forms of knowledge [11].

A triangulation of methods suggests that a belief of schizophrenia as unfamiliar and unpredictable is reflected in heightened arousals of HCNV emotions and facial muscle activation at the levator labii superioris. Similarly, a belief of depression as relatively familiar and comprehensible is reflected in heightened arousals of LCPV emotions, although the line between compassion and pity is unclear (18).

This research also explored individual differences in appraisal. We found no moderating effects of disgust-sensitivity or quantity of prior contact. This may be because measurement of frequency of contact may simply not be appropriate for understanding perceptions of mental illness, given the subtlety of prohibitions around contact [10]. Moreover, there are social desirability biases that differentiate ‘perceived’ from ‘experienced’ contact. However, as the study was powered for main effects only, these findings are tentative, and call for further research.

The evidence from this study for an appraisal of mental illness as Other has its limits. One key issue is the cultural diversity in the sample. Studies of perceptions of mental health have long highlighted its sensitivity to context [6,9,48]. Unfortunately, in a sample of this size, it was not possible to pull apart cultural factors. We encourage future research to be sensitive to the cultural context in which beliefs and behaviours about mental health emerge and explore this within key groups. Additionally, this research is conducted in a student sample. We do not intend generalisability past student populations in the UK, a group who have been highlighted as important to target [12]. However, it is worth noting these beliefs of contamination were found in a student sample. This highlights the need to be careful not to equate mental health-related knowledge with a lack of mental health related stigma. Although student groups are recurrently found to have higher levels of biomedical mental health related knowledge compared to the general public [1,2] this highlights how knowledge and beliefs about mental illness are not interchangeable. Indeed, mental health professionals have recurrently been found to hold stigmatising beliefs about mental illness, especially for beliefs expressed implicitly [49].

Additionally, this study has methodological implications for the examination of health-related stigmas. It highlights the need for public health researchers to use ecologically relevant research instruments, which prioritize the embodied and affective processes involved in representation [46]. However, it also exposes some of the methodological difficulties this principle presents. For example, an open coding of the studies qualitative elements resulted in the removal of the studies two control conditions [44]. To respond to the difficulties, we encourage researchers to employ methods that allow critical evaluation throughout the research process, such as mixed-methods and triangulation- based designs [50].

## 5. Conclusions

This exploratory study suggests that mental illness continues to be constructed as Other. Its Otherness was experienced by participants as a threat to the Self, involving appraisals including disgust and fear, compassion and pity. Contact with this Otherness was experienced as risky and a violation of social norms prohibiting certain forms of intimate contact. To violate these social norms, was to risk contamination. By engaging in strategies that separate the Self from the Other, a self-perception as in-control was maintained, and the Otherness of mental illness reproduced.

This study’s re-engagement with the more symbolic and subtle prohibitions around contact with mental ill-health has implications for the design and evaluation of public anti-stigma campaigns. Namely, it highlights the need for public health professionals to consider the multiple possible forms of contact the public experiences, and in particular, the need to pay greater attention to these intimate forms of contact. Furthermore, given the arguable construction of mental illness as Other and contaminating, it may be instructive for public health practitioners to consider the effects previous campaigns have had on the public’s perceptions of sexually transmitted diseases, which the public appear to engage with through a similar image to that which they hold of mental illness [47]. For example, campaigns focusing on more positive themes of self-fulfillment and joy have been considered more successful than those that prioritise biomedical knowledge in limiting infection rates [46]. Drawing on this, it may be useful to focus on in service-user experience over biomedical differences [12]. This may include prioritising service-user voices in the design and evaluation of public health campaigns [12] and in particular, paying greater recognition to the multiple factors users define for themselves as recovery [50]. 

This study was exploratory in nature. Much remains unknown about the subjective experience of contact with mental illness, and how this may vary by contexts and culture. It is through understanding the public’s constructions of ‘Other-ness’, including their beliefs about contamination that we can start to challenge the stigmatisation of mental illness [10,11].

## Figures and Tables

**Table 1:** Participant Demographics.

Gender	Male	(13)
	Female	(23)
Age	Range	(18–44)
	Mean	(24)
	Standard deviation	(5)
Subject	Arts and Humanities	(11)
	Biological and Physical Sciences	(5)
	Mathematics and Engineering	(7)
	Psychological and Health Sciences	(5)
	Business and Management	(8)
Nationality	Western Europe (17/36)	Britain (11) Italy (2) France (2) Spain (2)
	Eastern Europe (7/36)	Poland (1) Czech Republic (1) Croatia (1) Slovakia (1) Bulgaria (1) Romania (1) Russia (1)
	Asia (10/36)	China (4) Thailand (3) Malaysia (2) Taiwan (1) Singapore (1)
	Americas (2/36)	Canada (1) Brazil (1)

**Table 2:** Self-Reported Prior Contact with Mental Illness.

Frequency of Prior Contact with Mental Illness	N (%)
No prior contact with mental illness	8 (22.2)
One or less forms of prior contact with mental illness	17 (47.2)
Two or less forms of prior contact with mental illness	29 (80.6)
Three or less forms of prior contact with mental illness	34 (94.4)
Four or less forms of prior contact with mental illness	36 (100)

**Table 3:** Forms of Prior Contact with Mental Illness.

Reported and Intended Behaviour Scale (RIBS) Question	N (%)
Are you currently living, or have ever lived with, someone with a mental health problem?	14 (39)
Are you currently working, or have ever worked with, someone with a mental health problem?	21 (42)
Do you currently have, or have you ever had, a neighbour with a mental health problem?	10 (36)
Do you currently have, or have you ever had, a close friend with a mental health problem?	17 (47)

**Table 4:** Appraisal of Contact with Depression and Schizophrenia.

Dependent Variable	Mediator	df(test)	df(error)	F	*p*	η_p_^2^
HCPV		1.00	35.00	0.574	0.712	0.004
LCPV		1.00	35.00	17.513	<0.001 **	0.361
	DSQ	1.00	34.00	1.121	0.298	0.035
	PCMI	1.00	34.00	1.096	0.303	0.034
LCNV		1.00	35.00	<0.001	0.992	<0.001
HCNV		1.00	35.00	5.264	0.028 *	0.141
	DSQ	1.00	34.00	3.620	0.066	0.102
	PCMI	1.00	34.00	0.031	0.862	0.001
Levator Labii Activation		1.00	35.00	5.037	0.032 *	0.140
	DSQ	1.00	34.00	0.421	0.521	0.013
	PCMI	1.00	34.00	0.029	0.521	0.001

* = *p* < 0.05; ** = *p* < 0.01.

## Appendix A: Vignettes:

### Appendix A.1. Depression

#### Appendix A.1.1. Side 1:

**Please take a couple sips of water and hold the water in your mouth**

When you are ready, please turn over the piece of paper, and Press F1 on the keyboard.

#### Appendix A.1.2. Side 2:

Firstly, please close your eyes and imagine that you have been assigned to work on a class project with someone you don’t know well. However, your friend has told you that they know your partner has been diagnosed with depression.

Now imagine that the two of you are sitting in the cafeteria taking a break from work. You are both drinking a cup of tea, when you realise you have picked up the wrong mug, and have been drinking from the one your classmate has already used. Imagine that water in your mouth comes from their mug.

Please reflect on what feelings you experienced when you imagined you had drunk from the mug your classmate had already used, and fill out the emotion-wheel presented in front of you.

Once this is complete please press F2 on the key board, and swallow the water.

### Appendix A.2. Schizophrenia

#### Appendix A.2.1. Side 1:

**Please take a couple sips of water and hold the water in your mouth**

When you are ready, please turn over the piece of paper, and Press F1 on the keyboard.

#### Appendix A.2.2. Side 2:

Firstly, please close your eyes and imagine that you have been assigned to work on a class project with someone you don’t know well. However, your friend has told you that they know your partner has been diagnosed with schizophrenia.

Now imagine that the two of you are sitting in the cafeteria taking a break from work. You are both drinking a cup of tea, when you realise have picked up the wrong mug, and have been drinking from the one your classmate has already used. Imagine that water in your mouth comes from their mug.

Please reflect on what feelings you experienced when you imagined you had drunk from the mug your classmate had already used, and fill out the emotion-wheel presented in front of you.

Once this is complete please press F2 on the key board, and swallow the water.

### Appendix A.3. Common Cold

#### Appendix A.3.1. Side 1:

**Please take a couple sips of water and hold the water in your mouth**

When you are ready, please turn over the piece of paper, and Press F1 on the keyboard.

#### Appendix A.3.2. Side 2

Firstly, please close your eyes and imagine that you have been assigned to work on a class project with someone you don’t know well. However, your friend has told you that they know your partner has a cold.

Now imagine that the two of you are sitting in the cafeteria taking a break from work. You are both drinking a cup of tea, when you realise have picked up the wrong mug, and have been drinking from the one your classmate has already used. Imagine that water in your mouth comes from their mug.

Please reflect on what feelings you experienced when you imagined you had drunk from the mug your classmate had already used, and fill out the emotion-wheel presented in front of you.

Once this is complete please press F2 on the key board, and swallow the water.

### Appendix A.4. No Added Medical Description

#### Appendix A.4.1. Side 1:

**Please take a couple sips of water and hold the water in your mouth**

When you are ready, please turn over the piece of paper, and Press F1 on the keyboard.

#### Appendix A.4.2. Side 2:

Firstly, please close your eyes and imagine that you have been assigned to work on a class project with someone you don’t know well.

Now imagine that the two of you are sitting in the cafeteria taking a break from work. You are both drinking a cup of tea, when you realise have picked up the wrong mug, and have been drinking from the one your classmate has already used. Imagine that water in your mouth comes from their mug.

Please reflect on what feelings you experienced when you imagined you had drunk from the mug your classmate had already used, and fill out the emotion-wheel presented in front of you.

Once this is complete please press F2 on the key board, and swallow the water.

## Appendix B: Interview Guide

- Could you tell me a bit about how you felt imagining using the same mug as the classmates described.
  - Probe: what was the sensation of having the water in your mouth? How comfortable did it feel? Which sort of emotions were you feeling?
- Did it feel the same each time?
  - Probe: Where there any differences? Was the intensity of your feelings the same each time? Was there one test that was particularly uncomfortable for you?
- Was there anything you were worried about?
  - Probe: What sort of ideas might you have about why that was?
- Do you think there any sort of things people might be uncomfortable doing if they knew someone with mental health problems had already used the same object?
  - Probe: Which sort of actions? Which sort of objects? Around which sort of people? Why do you think that might be?
- Is there anything else more you want to say?
  - Probe: Are there any questions you think I should have asked you?
